# Rat Model of Parkes Weber Syndrome

**DOI:** 10.1371/journal.pone.0133752

**Published:** 2015-07-28

**Authors:** Krzysztof Bojakowski, Gabriela Janusz, Iwona Grabowska, Oliwia Zegrocka-Stendel, Agnieszka Surowiecka-Pastewka, Magdalena Kowalewska, Dorota Maciejko, Katarzyna Koziak

**Affiliations:** 1 Department of Immunology, Biochemistry and Nutrition, Medical University of Warsaw, Warsaw, Poland; 2 Department of Internal Medicine, Hypertension and Vascular Diseases, Medical University of Warsaw, Warsaw, Poland; 3 Department of Cytology, Faculty of Biology, University of Warsaw, Warsaw, Poland; 4 Department of Molecular and Translational Oncology, Maria Sklodowska-Curie Memorial Cancer Center and Institute of Oncology, Warsaw, Poland; Oklahoma State University, UNITED STATES

## Abstract

The Parkes Weber syndrome is a congenital vascular malformation, characterized by varicose veins, arterio-venous fistulas and overgrown limbs. No broadly accepted animal model of Parkes Weber syndrome has been described. We created side-to-side arterio-venous fistula between common femoral vessels with proximal non-absorbable ligature on common femoral vein limiting the enlargement of the vein diameter in Wistar rats. Contralateral limb was sham operated. Invasive blood pressure measurements in both iliac and inferior cava veins were performed in rats 30 days after fistula creation. Tight circumference and femoral bone length were measured. Histopathology and morphology of *soleus* muscle, *extensor digitorum longus* muscle, and the common femoral vessel were analyzed. 30 days following arterio-venous fistula creation, a statistically significant elevation of blood pressure in common iliac vein and limb overgrowth was observed. Limb enlargement was caused by muscle overgrowth, varicose veins formation and bone elongation. Arterio-venous fistula with proximal outflow limitation led to significant increase of femoral vein circumference and venous wall thickness. Our study indicates that the described rat model mimics major clinical features characteristic for the human Parkes Weber syndrome: presence of arterio-venous fistula, venous hypertension and dilatation, varicose veins formation, and the limb hypertrophy. We reveal that limb overgrowth is caused by bone elongation, muscle hypertrophy, and venous dilatation. The newly established model will permit detailed studies on the mechanisms underlying the disease and on the efficacy of novel therapeutic strategies for the Parkes Weber syndrome treatment.

## Introduction

The Parkes Weber syndrome (PWS), first described in 1907 [1], is characterized by a triad of arteriovenous fistulas (AVF), varicose veins and bone and soft tissue hypertrophy leading to limb enlargement. The presence of AVF distinguishes PWS from the Klippel-Trenaunay syndrome, known also as the capillary-lymphatic-venous malformation, CLVM [2]. The symptoms of PWS are congenital and present at birth. Most cases of PWS are sporadic, although familial histories have been reported. Vascular anomalies usually affect a limb, most commonly a leg, and less often a trunk. Capillary malformations, forming geographic patterns, are typically located on lateral side of the limb, buttocks or trunk. The appearance of varicose veins and dilated superficial veins in older age is triggered by arteriovenous shunt, venous hypertension and insufficiency of the deep venous system. The enlargement of a limb is present at birth, and the axial overgrowth can enlarge in postnatal period [3, 4]. Cases of shortened lower extremity and pelvis malformations have also been described [5]. Arteriovenous shunt may also lead to cardiac system failure or to limb ischemia. Not surprisingly, PWS, similarly to other vascular malformations, significantly reduces the quality of life of the affected patients [6].

Diagnosis of PWS is mostly based on color Doppler ultrasound, computer tomography, magnetic resonance imaging and rarely on arteriography all of which permit to detect and characterize vascular malformations such as AVF. The treatment of patients with PWS is mainly symptomatic. Compression therapy is used to reduce symptoms of chronic venous insufficiency and lymphatic edema. In selected cases invasive procedures are performed. Surgical treatment is difficult and may require several intravascular procedures, such as embolization, sclerotherapy or classic open operations involving arteriovenous fistula ligation. In severe cases of ischemic extremities amputation is the only therapy.

It has been documented that in a majority of PWS patients vascular malformations result from loss-of-function mutations in the *RASA1* gene encoding p120 Ras GTPase-activating protein [7, 8]. Although there has been no mechanistic explanation of how the mutations of a globally expressed gene specifically affect vasculature, a recent work suggests that deregulation of EPHB4/RASA1/mTORC1 signaling in endothelial cells caused by insufficient RAS inactivation could disrupt the process of arteriovenous differentiation, creating malformations in capillaries, arteries and veins [9]. In addition, there is at least one documented case of PWS patient demonstrating lymphatic abnormalities probably resulting from mispatterning of peripheral and mesenteric lymphatics and insufficiency of hypertrophic lymphatic channels [7]. This remains in concordance with data obtained from RASA1-knock-out mice which exhibited hyperplasia of the initial lymphatics as well as dilation of initial and collecting lymphatic vessels [7].

To date, no animal model reproducing complex manifestations of PWS and applicable for research on etiology, treatment and prevention of the disease, has been developed. The only attempt reported to date in mammals was generation of Rasa1-deficient mice [10]. Such animals develop lymphatic malformations which are also observed in PWS patients, but other abnormalities characteristic for the disease, *e*.*g*. vein anomalies, limb overgrowth, are not described. Therefore, there is an ever-existing need to provide an animal model of PWS, which would reflect the pathophysiological characteristics of PWS and be an effective research tool in etiopathogenesis and pharmacological studies. As such, it would permit the evaluation of the disease course, and the assessment of potential effects of novel therapeutic modalities to prevent, treat, delay or ameliorate the disease symptoms.

The aim of the study was to create a mammalian model of multi-symptomatic PWS which would permit to investigate the possible mechanisms underlying the disease and facilitate the search for pharmacological solutions and invasive therapy of PWS. Our results indicate that the described rat model recapitulates major clinical features characteristic for the human PWS: presence of AVF, venous hypertension and dilatation, varicose veins formation, and the limb hypertrophy.

## Materials and Methods

For all the experiments 6–10 week-old male Wistar rats were used. The experimental procedures were reviewed and approved by the Bioethics Commission of Medical University of Warsaw (No. 46/2012), where the animal experiments were performed.

### Animal model of PWS—technique description

After induction of general anesthesia with 75 mg of ketamine (Pfizer, Poland) and 5 mg of midazolam (Polfa, Poland) per kg i.p., longitudinal skin incision just below inguinal ligament was performed. Common femoral vessels were dissected from surrounding tissues proximally to the orifice of superior epigastric vessels. After application of micro-vascular clamps on common femoral vessels longitudinal 2 mm long incisions on adherent artery and vein were performed. Afterwards, side-to-side anastomosis between common femoral vessels was created to generate arteriovenous fistula (AVF) using non-absorbable monofilament suture (10/0). Subsequently, after removal of micro-vascular clamps, AVF patency was checked and hemostasis was achieved. To avoid lung edema, increase of blood inflow to inferior cava vein was limited by application of non-absorbable ligature of 0.9 mm internal diameter. Mainly distal blood flow caused by this ligation generated venous hypertension while mitigating the consequences of right ventricular heart failure. The resultant vein lumen corresponded to approximately 80–90% of the initial vein lumen. Next, subcutaneous tissue and skin were sutured with absorbable suture. Sham operation on control limb included skin incision, common femoral vessels dissection, and subcutaneous and skin suture. All the subsequent analyzes were performed 30 days after AVF creation.

### Blood pressure measurement

After induction of general anesthesia, a catheter connected to Millar pressure transducer was introduced into a right lumbar vein. Inferior cava vein and both common iliac veins (proximal to the limitation ligature)—one with AVF, and the other without AVF (sham-operated), were selectively cannulated and invasive blood pressure measurement was performed. Data were collected and analyzed with PowerLab monitoring and acquisition system (ADInstruments, UK), LabChart software (ADInstruments, UK). Venous pressures were presented as centimeters of water (cmH_2_O).

### Tight circumference, skin, muscle, common femoral vessel, femoral bone procurement, muscle weight, femoral bone length measurements

Following invasive blood pressure measurements, the animals were sacrificed by barbiturate overdose (100 mg/kg, i.p.; Biowet, Poland). After the euthanasia, measurements of tight circumference of both (*i*.*e*., with AVF and sham-operated) limbs were performed and samples of the following tissues were procured for further analyses: femoral vessels with adjacent proximal segments of femoral superficial vessels, soleus muscles (SOL) and the *extensor digitorum longus* muscles (EDL). Dissected muscles were weighted. Additionally, femoral bones were also collected and their length measured with electronic slide clipper.

### Histopathological examination and morphometric analyses—muscles, femoral vessels

The samples of femoral vessel and the dissected muscles were frozen by placing them for several seconds in 2-methylbutane (Sigma-Aldrich, Poland) cooled in liquid nitrogen, transferred to liquid nitrogen and stored at -80°C. Tissues were cut frozen using a cryostat (Microm HM 505, Thermo Fisher Scientific, Germany) into 10 μm-thick sections, mounted on the glass slides for 30 minutes in 37°C and allowed to dry. Next, the slides were placed in phosphate buffered saline (PBS) for 15 minutes and then stained for 40 minutes with Harris’s hematoxylin (Sigma-Aldrich). After washing with tap water, the slides were stained with eosin Y (Sigma-Aldrich) for 3 minutes. Specimens were washed again with water and then mounted using UltraMount (DakoCytomation, Denmark). For Masson's Trichrome (Sigma-Aldrich) stains the specimen were first stained with Harris’s hematoxylin and washed as described above, then rinsed in deionized water and stained in Biebrich Scarlet Acid Fuchsin solution for 5 minutes, according to manufacturer instructions. Following a rinse in deionized water and 5-minute treatment with phosphotungstic-phosphomolybdic acid, slides were transferred into Aniline Blue solution and stained for 5 minutes. Next, specimens were placed in 1% acetic acid solution for 2 minutes, rinsed in distilled water, and mounted using UltraMount.

The examined sections were viewed under the light microscope (Nikon Eclipse TE200; Nikon Instruments, Inc., Japan) under the 4x magnification lens and photographed using the NIS Elements F 2.30 software (Nikon Instruments, Inc., Japan) at the same resolution (2560 x 1920 pixels).

Morphometric analyses of the sections were performed using the Adobe Photoshop CS5 Extended software (version 12.0 x32). Fiber numbers per view (1.25 x 1.25 mm field of view / 1500 x 1500 pixels per image) and fiber areas within individual muscles sections were measured. Histopathology of femoral veins was assessed and this included vein circumference as well as venous wall thickness measurements.

### Statistical analysis

The results were expressed as arithmetic means and standard errors of the mean (SEM). Comparisons of the results obtained for limbs with AVF, sham-operated and control limbs were performed using the paired t-test (GraphPad Prism v.6.01). The differences of p<0.05 were considered statistically significant.

## Results

### Animals

All animals survived throughout the duration of the experiment and did not manifest signs of chylothorax or chyloascites.

### Blood pressure

**Fig 1:**
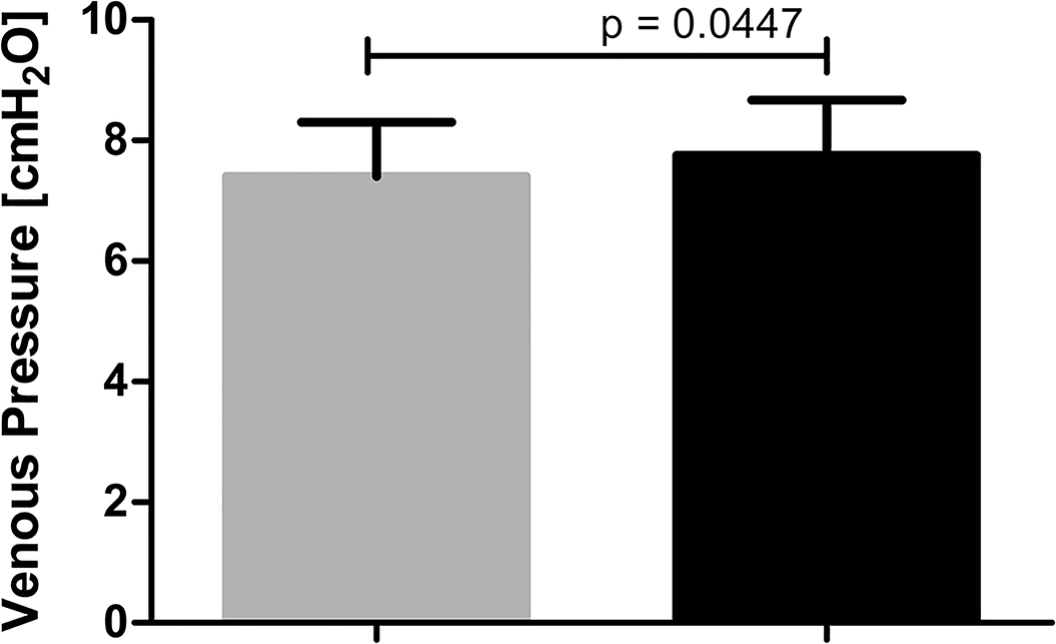
Venous pressure in sham-operated limbs (grey column, n = 13) and limbs with AVF (black column, n = 13).

Creation of femoral AVF with proximal outflow limitation induced an increase of venous pressure in a limb. Mean blood pressure measured in common iliac vein 30 days after AVF creation was statistically significantly higher in the limbs with AVF than in sham-operated limbs (7.76±0.915 and 7.41±0.899 cmH_2_O, respectively; p = 0.04) (Fig 1).

### The histopathological analysis of the venous vessels

**Fig 2:**
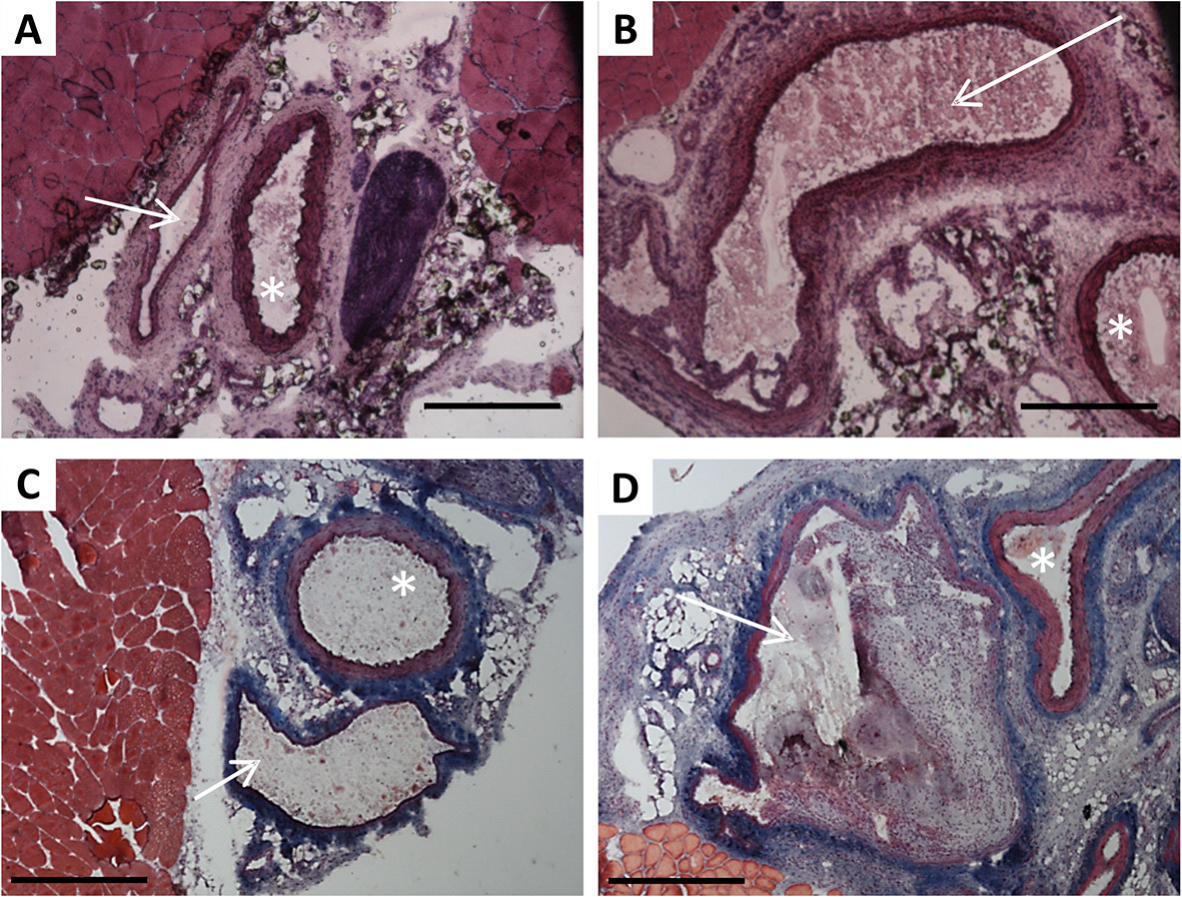
Femoral blood vessels morphology () in sham-operated limbs (A, C) and limbs with AVF (B, D). Panels A, B—hematoxylin and eosin stained cryosections; panels C, D—Masson’s trichrome stained cryosections. White arrow indicates the vein, asterisk–the artery. Scale bar: 500μm

**Fig 3:**
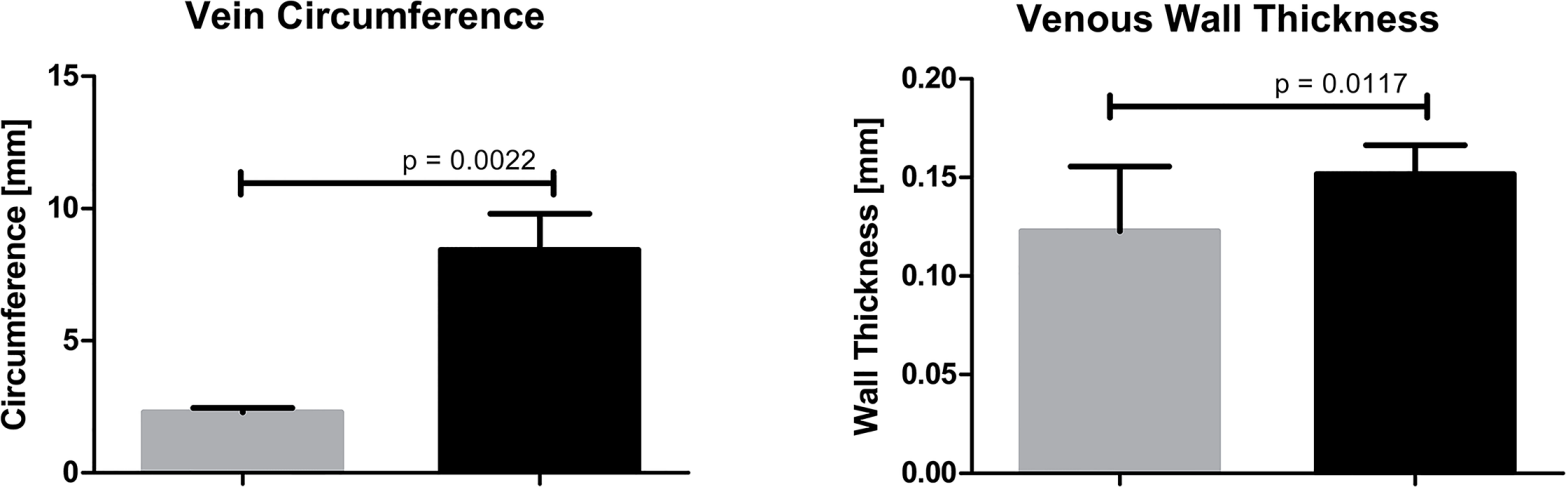
Morphometric analysis of lower limb veins—difference between sham-operated limbs (grey columns, n = 9) and limbs with AVF (black columns, n = 9).

AFV with proximal outflow limitation leads to significant changes in vein morphology, as demonstrated in Fig 2. Femoral vein circumference was significantly increased in limbs with AVF compared to sham-operated limbs (8.43±1.37 *vs*. 2.29±0.17 mm, p = 0.0022). It was concomitant with significant increase of venous wall thickness 0.15±0.015 *vs* 0.12±0.033 mm, p = 0.017) (Fig 3). AVF caused considerable vein dilatation (Figs 2 and 3) and varicose veins formation (data not shown).

### Procurement of the muscles, common femoral vessels and femoral bones. The tight circumference, muscle weight and femoral bone length measurements

**Fig 4:**
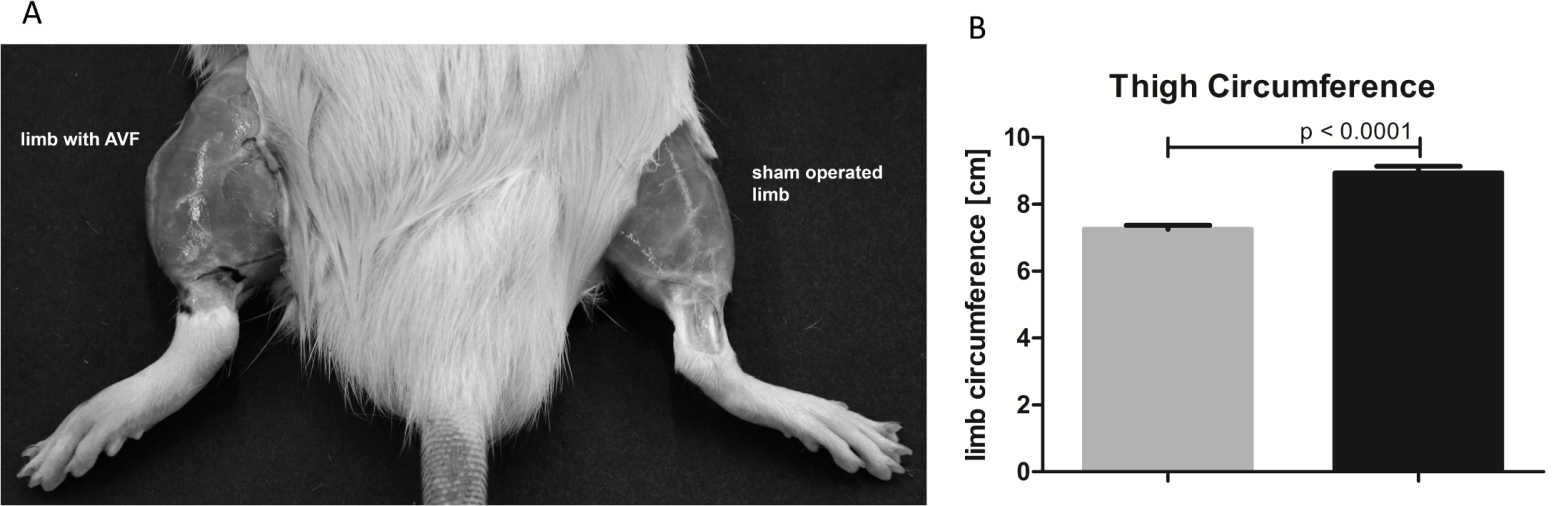
Limb enlargement (panel A) and tight circumference of sham-operated limbs (grey column, n = 14) and limbs with AVF (black column, n = 14) measurement (panel B).

Creation of AVF with proximal ligation initiated progressive and significant limb overgrowth, and 30 day after operation tight circumference in limbs with AVF was significantly bigger than in control sham-operated limbs (8.94±0.202 *vs*. 7.26±0.114 cm, respectively; p < 0.0001) (Fig 4). Noteworthy, the limb enlargement did not occur immediately following the AVF creation as a result of limb swelling.

**Fig 5:**
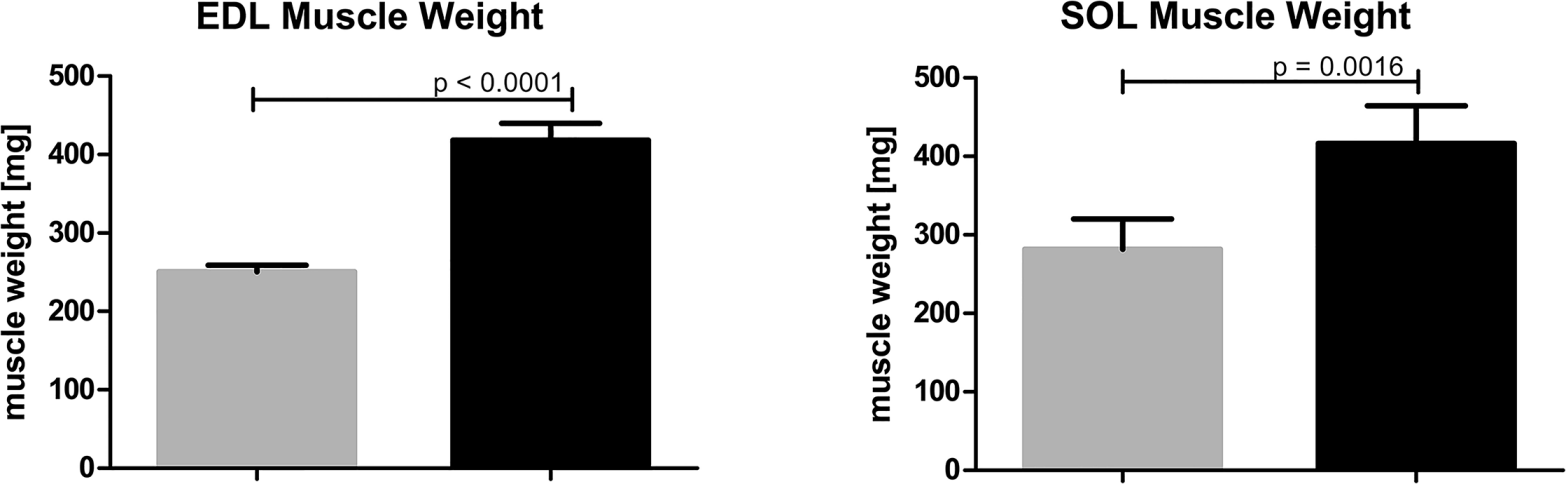
Muscle overgrowth in hind limb with AVF—difference in EDL and SOL muscles weights of sham-operated limbs (grey columns, n = 14) and limbs with AVF (right columns, n = 14).

**Fig 6:**
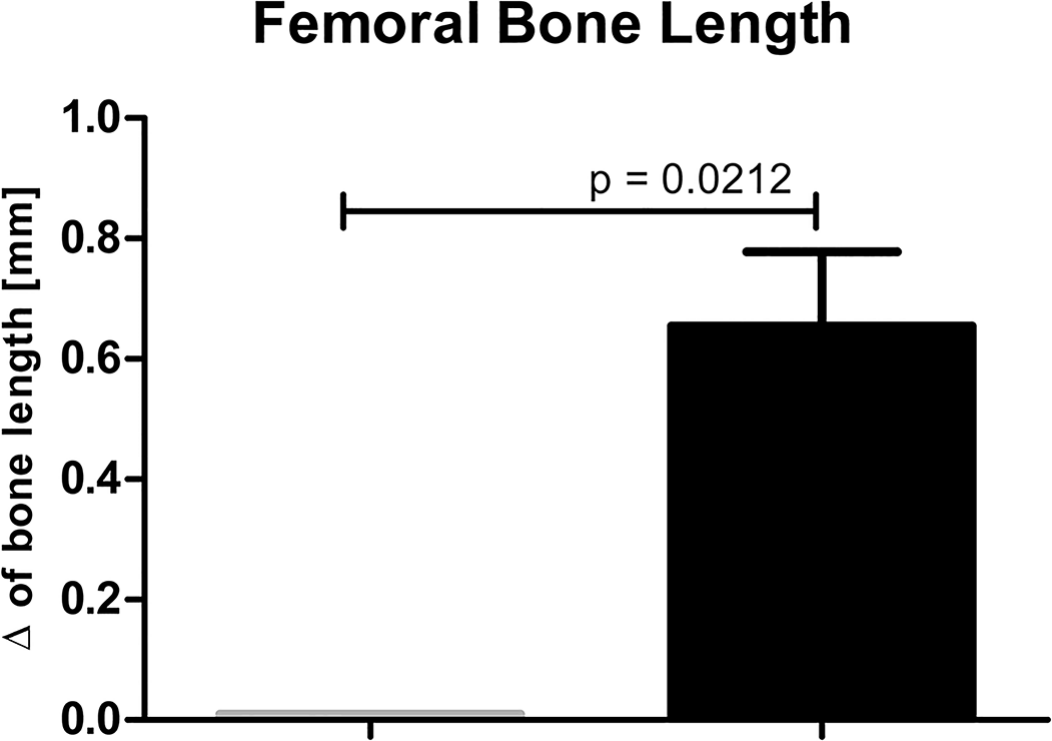
Difference in bone lengths in non-operated animals (i.e. the value of the length of the right femoral bone subtracted from the left femoral bone; left grey column, n = 3 bone pairs), and operated animals (i.e., value of the length of femoral bone of sham-operated limb subtracted from the length of femoral bone of the limb with AVF, n = 11 bone pairs).

Limb enlargement triggered by AVF was concomitant not only with venous widening but also with the limb muscles overgrowth. Muscles isolated from limbs with AVF were significantly heavier than in sham-operated group. Specifically, the muscle weight values for EDL in limbs with AVF compared to sham-operated limbs were 418.23±21.41 *vs*. 250.64±30.84 mg, respectively (p < 0.001) and for SOL—416.50±47.98 *vs*. 281.86±38.15 mg, respectively (p = 0.0016) (Fig 5). The limb overgrowth was also associated with the elongation of femoral bones. There was no difference in femoral bones’ length of control, un-operated animals, while in the operated animals femoral bones isolated from limbs with AVF were significantly longer compared to those isolated from sham-operated limbs *i*.*e*., 0.01±0.00 mm *vs*. 0.65±0.12 (p = 0.0212) (Fig 6).

### The histopathological analysis of SOL and EDL muscles

**Fig 7:**
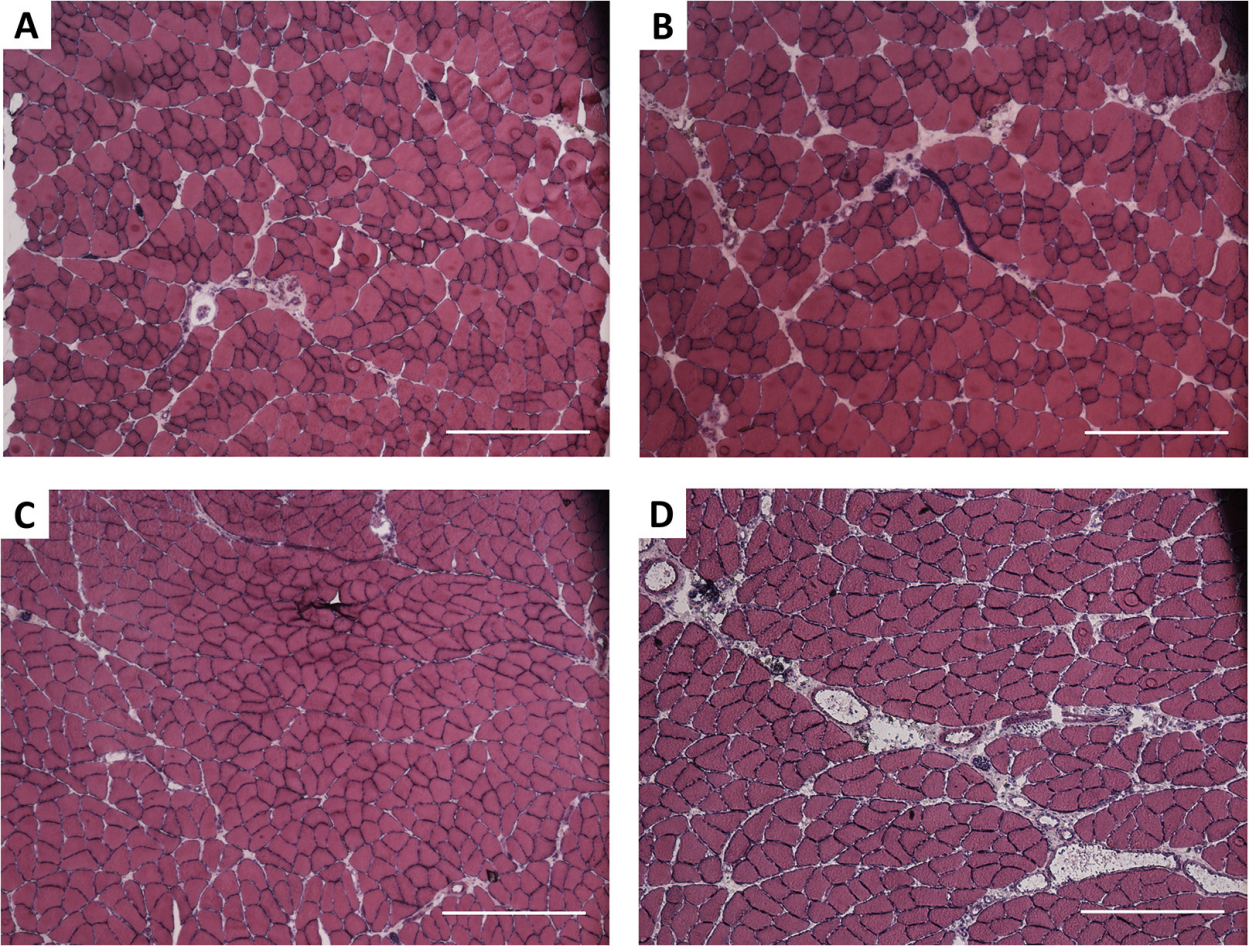
EDL and SOL muscles morphology. Hematoxylin-eosin-stained cryosections of EDL (A, B) and SOL (C, D); muscles from sham-operated limbs (left column; A, C) and limbs with AVF (right column; B, D). Scale bar: 500μm

**Fig 8:**
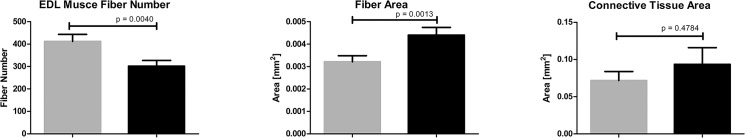
Number and area of muscle fibers, and connective tissue area in EDL muscle. A difference between sham-operated (grey column; n = 9) and limbs with AVF (black column; n = 11).

As shown in Fig 7, the muscles overgrowth was concurrent with histopathological changes in their structure. Morphometric quantification of this observation (Fig 8) revealed that in EDL muscles AVF creation led to a significant decrease in muscle fiber number (301±25.6 *vs*. 412±31.6 in limbs with AVF and sham-operated limbs, respectively, p = 0.0040) and an increase in fiber area (4403±339.8 *vs* 3206±275.4 μm^2^ in limbs with AVF and sham-operated limbs, respectively, p = 0.0013). We did not observe a significant influence of our procedure on connective tissue in overgrown EDL muscles. Differences in connective tissue areas in EDL muscles of limbs with AVF and sham-operated limbs were insignificant, *i*.*e*., 0.0936±0.0222 mm^2^ *vs*. 0.0717±0.0121 mm^2^ (p = 0.4784), respectively.

**Fig 9:**
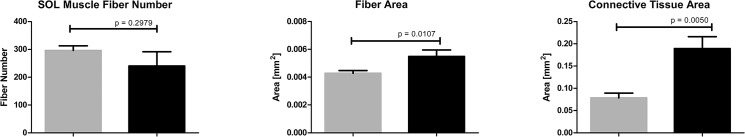
Number and area of muscle fibers and connective tissue area in SOL muscle. A difference between sham-operated (gray column, n = 12) and limbs with AVF (black column, n = 12).

AVF creation induced significant changes also in SOL muscles. Although the number of muscle fibers did not differ significantly between limbs with AVF and sham-operated limbs (240.6±50.97 *vs*. 295.8±17.07, respectively, p = 0.2979), the fiber area (5481±466.8 *vs*. 4274±205 μm^2^, p = 0.0107) was significantly increased in limbs with AVF in comparison with sham-operated limbs (Fig 9). While the creation of AFV did not considerably affect the connective tissue of EDL muscles, it caused a significant increase in the connective tissue area in SOL muscles (0.189±0.027 *vs*. 0.078±0.011 mm^2^, p = 0.0050).

Although the muscle morphology assessment did not include extensive analysis of lymphatic vessels density, their count increased at least two-fold in both EDL and SOL muscles in limbs with AVF compared to sham-operated limbs (data not shown).

## Discussion

The study provides a description of the first mammalian model of multi-symptomatic PWS. We demonstrate that in rats, creation of 2 mm fistula between common femoral artery and common femoral vein with vein ligature limits proximal outflow and induces the occurrence of symptoms characteristic for PWS. The reported surgical procedure causes venous hypertension and thickening of venous wall, varicose veins formation and the limb hypertrophy manifested with the increase in the tight circumference, elongation of the femoral bones, and hind limb muscles hypertrophy. It must be noted that apart from missing the genetic factor underlying the disease (OMIM Entry ID: 608355), this rat model is based on a single AVF instead of multiple vascular malformations observed in PWS patients. However, the presence of only one fistula significantly simplifies the observations of the changes in haemodynamic response and muscle or bone alternations. Additionally, multiple fistulas would practically disable the establishment and control of desired blood flow through shunts—high enough to cause vessel and musculoskeletal changes but low enough to prevent ischemic steel syndrome and heart failure caused by decompensated circulation resulting from high volume arterio-venous leakage.

To date, the lack of animal models of PWS have significantly impaired the efforts to discern relevant pathophysiological mechanisms and posed a major problem in the search for pharmacological solutions and invasive therapy of the disease. Creation of AVF and a ligature in young adult rats as described in this study will allow to investigate the mechanisms leading to PWS that develop both in prenatal and postnatal period as consequences of vessel malformations. AVF should be localized distally from the ligature, far enough to not disturb blood flow in anastomosis. There should be no branch (collateral vein) between the ligature and the fistula, as it would influence blood pressure and lead to blood steal. Ligature should preserve vein lumen and prevent its dilatation. An invariable diameter of the outflow vein coexistent with an increase in venous inflow rate via AVF (due to relative venous outflow obstruction resulting from increased blood flow) ultimately leads to an increase in peripheral venous pressure. The constriction of blood flow is necessary to prevent rat’s death resulting from heart failure induced by severe arterio-venous leakage. Contralateral, sham operated limb may serve as a control.

The progression and severity of symptoms characteristic for PWS in our model depend on many factors, of which the two most important include the size of AVF determining the blood flow through the fistula, arteries and veins, particularly those localized distally from the fistula and the size of the proximal ligature, which determines the blood flow rate and blood pressure distribution. Additionally, the outcome might be influenced by the age of animals, as well as the time of observation following the AVF creation.

Histopathological alterations observed in our model result from progressive processes induced with generation of AVF creation. It has been well established that vasodilation, vascular remodeling and varicose veins formation, all of which are commonly observed in PWS patients, result from the presence of AVF [11]. While this distinct underlying cause of PWS has been already well recognized and understood, its relationship to tissue hypertrophy has remained obscure [12]. Significant weight augmentation of both analyzed hind limb muscles—EDL and SOL, reaching approximately 160% of sham-operated limb muscle weight, was paralleled with considerable alteration of their structure. Although not all of the detected differences reached statistical significance, in both muscles a decrease in muscle fiber number, an increase in fiber area, and an expansion in connective tissue area were recognized. All the observed changes are typical for regenerating muscles, which suggests that an episode of muscle injury, most probably triggered by ischemia and venous hypertension following the AVF creation, must have occurred. Certain dissimilarities in EDL and SOL responses to AVF induced injury, *i*.*e*. more pronounced decrease in the number of fiber muscles in EDL *vs*. SOL, may reflect functional, biochemical, and morphological differences between skeletal muscles of different fiber type content. It has been previously shown that there are differences in the regenerative response of fast twitch EDL and slow twitch SOL to diverse types of injury, *e*.*g*. injection of anesthetic [13, 14] or crush [15]. In crush induced regeneration model, EDL muscle regenerates properly and completed reconstruction at day 14 after injury [15]. In contrast, SOL muscle undergoes fibrosis after crush injury and denervation [15–17]. Although connective tissue fibrosis is characteristic of regenerating muscle, maintaining its structural and functional integrity, excessive fibrosis may obstruct the process of tissue repair [18]. Statistically significant increase in connective tissue area in SOL muscles following AVF creation may therefore reflect an inefficient, ineffective and incomplete regeneration.

Interestingly, significant overgrowth of limb muscles following the AVF establishment resembles muscle hypertrophy after low-intensity training with blood flow restriction observed in humans [19]. Elevation in muscle protein synthesis correlated with mTOR complex 1 signaling activation and enhancement of translation initiation were suggested as possible mechanisms underlying this phenomenon [20, 21]. Moreover, exercise with blood flow restriction of limb muscle triggers a significant increase in plasma concentration of growth hormone (skeletal muscle growth promoting factor) [22]. It has been also speculated that an increase in muscle size after exercise of blood flow-restricted muscles results from the plasma/fluid shift from the vessels into the muscles [23]. The results of our study suggest that similar response of skeletal muscle tissue in the presence of blood flow restriction is caused by AVF.

Blood flow restriction may also be considered as a mechanism underlying elongation of femoral bone that was observed in the presence of AVF. The influence of blood flow on bone adaptation remodeling has been well recognized (reviewed in [19]). The current understanding of the link between venous occlusion and augmenting bone mass points toward increased intramedullary pressure and interstitial fluid flow as well as ischemia induced over-expression of several growth factors which may play a role in bone elongation. Activation of hypoxia inducible transcription factor (HIF) and, consequently, of vascular endothelial growth factor (VEGF), which are essential for bone remodeling [24–26] provide a connection between hypoxia, angiogenesis and osteogenesis vital for bone and joint development and growth. Additionally, hind limb ischemia influences mobilization and recruitment of bone marrow stem/progenitor cells [27].

Noteworthy, although still a preliminary finding, is the hyperplastic response of lymphatic system observed in the limb with AVF. Given the functional relationship between the lymphatic and blood vasculatures reflected in high incidence of lymphatic malformations in patients with congenital vascular malformations [28] including PWS [7], our results further strengthen the value of the newly established animal model.

Our results reveal that limb overgrowth commonly observed in patients with PWS is probably a consequence of tissue hypertrophy triggered by ischemia and venous hypertension. We hypothesize that limb enlargement triggered by presence of AVF results from multiple factors including muscle overgrowth, bone elongation, vein dilatation and varicose vein formation and lymphatic hyperplasia.

## Conclusions

The proposed rat model of PWS recapitulates of all major symptoms characteristic for PWS: venous hypertension and dilatation, varicose veins formation and the limb hypertrophy. The study demonstrates that limb overgrowth triggered by AVF is caused by femoral bone elongation, muscle hypertrophy and venous dilatation. The newly established model would permit to investigate the possible mechanisms underlying the disease and facilitate the search for pharmacological solutions and invasive therapy of PWS. In particular, the described model would enable the studies aiming at diminishing musculoskeletal consequences of hemodynamic changes triggered by AVF.
